# How can we teach medical students to choose wisely? A randomised controlled cross-over study of video- versus text-based case scenarios

**DOI:** 10.1186/s12916-018-1090-y

**Published:** 2018-07-06

**Authors:** Sascha Ludwig, Nikolai Schuelper, Jamie Brown, Sven Anders, Tobias Raupach

**Affiliations:** 1 Department of Cardiology and Pneumology, Göttingen University Medical Centre, Robert-Koch-Straße 40, D-37075 Göttingen, Germany; 2 Department of Haematology and Medical Oncology, Göttingen University Medical Centre, Robert-Koch-Straße 40, D-37075 Göttingen, Germany; 3 Health Behaviour Research Centre, University College London, 1-19 Torrington Place, London, WC1E 7HB UK; 4 Department of Legal Medicine, University Medical Centre Hamburg-Eppendorf, Butenfeld 34, D-22529 Hamburg, Germany

**Keywords:** Medical education, Clinical reasoning, Choosing wisely, Test-enhanced learning, Video

## Abstract

**Background:**

The *Choosing Wisely* campaign highlights the importance of clinical reasoning abilities for competent and reflective physicians. The principles of this campaign should be addressed in undergraduate medical education. Recent research suggests that answering questions on important steps in patient management promotes knowledge retention. It is less clear whether increasing the authenticity of educational material by the inclusion of videos further enhances learning outcome.

**Methods:**

In a prospective randomised controlled cross-over study, we assessed whether repeated video-based testing is more effective than repeated text-based testing in training students to choose appropriate diagnostic tests, arrive at correct diagnoses and identify advisable therapies. Following an entry exam, fourth-year undergraduate medical students attended 10 weekly computer-based seminars during which they studied patient case histories. Each case contained five key feature questions (items) on the diagnosis and treatment of the presented patient. Students were randomly allocated to read text cases (control condition) or watch videos (intervention), and assignment to either text or video was switched between groups every week. Using a within-subjects design, student performance on video-based and text-based items was assessed 13 weeks (exit exam) and 9 months (retention test) after the first day of term. The primary outcome was the within-subject difference in performance on video-based and text-based items in the exit exam.

**Results:**

Of 125 eligible students, 93 provided data for all three exams (response rate 74.4%). Percent scores were significantly higher for video-based than for text-based items in the exit exam (76.2 ± 19.4% vs. 72.4 ± 19.1%, *p* = 0.026) but not the retention test (69.2 ± 20.2% vs. 66.4 ± 20.3%, *p* = 0.108). An additional Bayesian analysis of this retention test suggested that video-based training is marginally more effective than text-based training in the long term (Bayes factor 2.36). Regardless of presentation format, student responses revealed a high prevalence of erroneous beliefs that, if applied to the clinical context, could place patients at risk.

**Conclusion:**

Repeated video-based key feature testing produces superior short-term learning outcome compared to text-based testing. Given the high prevalence of misconceptions, efforts to improve clinical reasoning training in medical education are warranted. The *Choosing Wisely* campaign lends itself to being part of this process.

**Electronic supplementary material:**

The online version of this article (10.1186/s12916-018-1090-y) contains supplementary material, which is available to authorized users.

## Background

One of the most important aims of undergraduate medical education is to enable future physicians to arrive at correct diagnoses and treatment recommendations based on the results of appropriate diagnostic tests. Making correct decisions is at least as important as avoiding unnecessary tests and incorrect diagnoses, and both need to be considered in the context of clinical reasoning [1]. Recently, the *Choosing Wisely* campaign has added another important feature to the professional approach to patient management [2, 3]. In order to avoid unnecessary or potentially harmful diagnostic and therapeutic procedures, recommendations compiled by medical societies in various countries across the globe [4] advise a more thorough consideration of the specific merits and limitations of these interventions and discourage the use of some in specific circumstances [5, 6]. Clinical reasoning abilities are a prerequisite for making ‘wise choices’ in clinical medicine, thus suggesting that teaching interventions aimed at fostering clinical reasoning may also enhance performance with regard to the recommendations derived from the *Choosing Wisely* campaign. Vice versa, these recommendations result from a stringent application of the principles of clinical reasoning. Thus, the choice of teaching formats that lend themselves to the discussion of these recommendations should be guided by a discussion of instructional formats used to address clinical reasoning itself. One effective approach to enhancing the complex cognitive functions related to clinical reasoning is case-based learning [7], which is traditionally facilitated in small groups [8] or at the bedside [9]. Given the limitations of these formats regarding resource intensity and availability of patients with rare but important diseases, computer-assisted case-based learning with virtual patients [10] has been proposed as a viable approach to teaching clinical reasoning [11, 12].

Digital interventions in medical education need to be informed by educational and psychological research. One recent example of this type of ‘translational research’ in medical education is the growing body of evidence showing that test-enhanced learning enhances knowledge and skills retention in undergraduate medical students. The basic idea is that questions can be used to stimulate learning processes [13, 14]. Thus far, a number of studies have shown that tests can in fact be used as learning tools in medical education [15]. For example, in one study involving 47 first-year medical students, repeated testing of factual knowledge in neurology elicited greater long-term retention than repeated study of the same material [16]. Another randomised study yielded a similar effect for the retention of practical (resuscitation) skills [17]. However, none of these studies focussed on clinical reasoning. One potential reason for this is that rote memory can easily be tested using multiple choice questions while testing clinical reasoning is more complex. One test format suited for this purpose is the so-called key feature question. A key feature is defined as a significant step in patient management. Key feature questions were designed to test the knowledge needed to avoid common errors occurring at these decisive points. For example, the key features in one of the cases used in this study referred to the management of a potentially life-threatening condition. First, students were prompted to provide the most likely diagnosis (key feature 1: pulmonary embolism) for a 44-year-old female patient who had been immobilised with lumbago for a week and who experienced sudden chest pain and shortness of breath following defaecation. In the second question, students were expected to identify calculation of the Wells Score as the next key feature in patient management. Following this, students were informed that this score was 6, and they were asked to name the most appropriate next step for the patient who was haemodynamically stable (key feature 3: CT scan). Upon confirmation of the diagnosis, students were asked about a diagnostic procedure that can inform risk stratification for this particular patient (key feature 4: PESI score, cardiac ultrasound or troponin measurement). In the fifth section of the case, the patient is no longer haemodynamically stable and requires catecholamine therapy, and students were asked to indicate the therapeutic action required (key feature 5: thrombolysis). A recent review [18] confirmed that key feature questions lend themselves to the assessment of clinical reasoning, and it has been suggested they could be used in a test-enhanced learning paradigm [19]. We recently reported the findings of a randomised study of repeated testing with key feature questions on outcomes related to clinical reasoning [20], wherein 6 months after the intervention, students retained significantly more content that had been presented in key feature questions compared to content that had been presented in a didactic format but without interspersed questions (56% vs. 49%; *p* < 0.001).

While this study showed that repeated case-based testing with key feature questions is more effective than repeated case-based study of the same material, the overall retention of procedural knowledge related to clinical reasoning at the 6-month follow-up was suboptimal. One reason might be that all the case-based material used in that study was presented as text on computer screens. While text can easily be scanned for relevant information, students might have found it harder to immerse in the clinical problem presented. This assumption is supported by psychological research dating back to 1975, when Godden and Baddeley demonstrated the importance of similarities between the learning environment and situations in which the learnt material needs to be recalled [21]. In addition, according to the dual-coding theory [22] and supporting original research [23], simultaneous presentation of audio-visual material is more likely to enhance retention of information than verbal material alone. Despite the increasing use of videos in undergraduate medical education, we are not aware of any studies addressing their value when used as part of exams focussing on clinical reasoning.

In summary, test-enhanced learning with video-based, computer-assisted exams could be an efficient way of enhancing student performance with regard to making the right choices about diagnostic tests and taking appropriate therapeutic action. In addition, the avoidance of unnecessary interventions as set out in the *Choosing Wisely* recommendations can ideally be addressed in key feature questions. To our knowledge, no previous study has reported student performance in this context. In addition, incorrect answers to key feature questions have rarely been analysed although they may reveal specific shortcomings in teaching that could be easily remedied.

This randomised cross-over study was designed to test the hypothesis that repeated testing with video-based key feature questions produces superior retention of procedural knowledge related to clinical reasoning compared to repeated testing with text-based key feature questions. The second aim was to investigate the effects of (non) alignment between presentation formats in the learning phase and subsequent exams on student performance. The third aim of this study was to identify common errors evident in student responses to key feature questions.

## Methods

### Study design

This was a randomised, controlled, non-blinded cross-over study involving fourth-year undergraduate medical students. At Göttingen Medical School, clinical reasoning is mainly addressed in study years 4 and 5. In addition to small-group case-based discussions and bedside teaching, students work on predefined patient case histories in computer-based ‘electronic seminars’ (e-seminars) lasting up to 45 min each. E-seminars are facilitated at the institution’s e-learning resource centre.

Students enrolled in the first term of the fourth year were eligible for study participation during the summer of 2015. During the first 14 weeks of term (‘learning phase’), students attended three thematic modules on cardiology, pulmonology, nephrology, rheumatology, haematology and oncology. Following stratification by sex and prior performance levels, they were randomised to one of two groups (A and B). Students in both groups attended weekly e-seminars during which they were presented with three patient case histories related to teaching content of the previous week. Case histories (‘virtual patients’) were adapted from an earlier study [20]; they had been designed according to a set of principles identified in a previous focus group study among German medical students [24]. Each patient case was broken up into five sections, and at the end of each section, students answered a key feature question on the most appropriate next diagnostic/therapeutic step by entering at least three letters in a box and choosing from a long menu of options provided by the exam software. An example of a key feature question is included in Additional file 1: Methods. Throughout this manuscript, the word ‘item’ refers to one key feature question, i.e. each case was made up of five items.

Case content was identical for all students but the format varied each week between ‘text cases’ or ‘video cases’. In text cases, content was presented on the computer screen in written format while dealing with video cases involved watching five short films (mean duration 68 s), and answering a question (i.e. one item) after each film. Films were scripted based on written cases that had been piloted in two consecutive student cohorts. Wherever possible, videos were closely aligned to text cases. However, inevitably, minor differences arose in the exact wording of content, which are fundamental to the difference between written information and audio-visual material showing a true interaction between a patient and a physician. For a sample video, please refer to Additional file 2.


**Additional file 2:** Sample video. Example of a video key feature item. This MP4-file is an example of a video key feature item featuring a 74 year-old simulated patient called “Mr. Teschke” (fictional name) with increasing shortness of breath. The interaction was scripted and both the patient and the doctor were played by actors who were not affiliated to the study team. Both individuals consented to participate in the research project and to be shown in this sample video. (MP4 7365 kb)


**Fig. 1:**
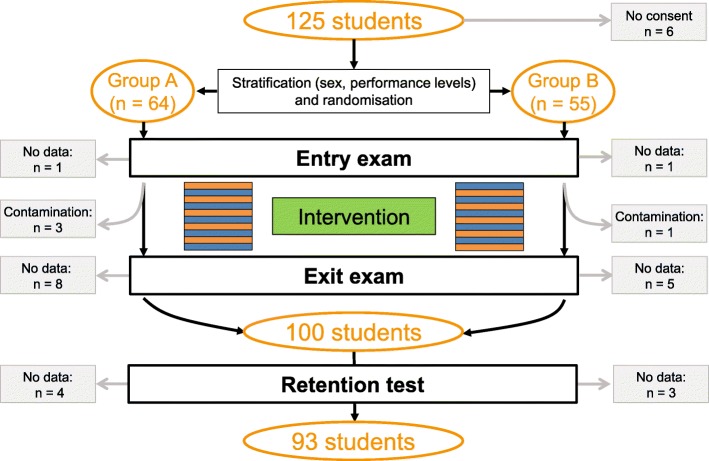
Flow of participants through the study. Orange boxes, video cases; blue boxes, text cases. Contamination occurred when students were erroneously exposed to the wrong presentation format (by reporting to the computer room assigned to the other group) at least once

During the learning phase, students in the two study groups were alternately exposed to video-based or text-based e-seminars with all students being exposed to the same content in any given week. For example, in week 2, students in group A worked on video cases (Fig. 1, orange boxes) while students in group B were exposed to text cases presenting the same content (Fig. 1, blue boxes). The presentation format then alternated systematically for each group across 10 sessions. No specific measures were taken to prevent students from discussing e-seminar content outside the sessions.

Given that there were three cases per week, each containing five key feature questions (i.e. items), students answered a total of 150 items during the learning phase. Of these, 14 items in group A were predefined as intervention items which meant that these items were presented as video-based questions. The same items were used as control items (i.e. text-based questions) in group B. Conversely, 14 intervention items for students in group B were used as control items in group A. Item assignment to the two study groups was balanced with regard to item difficulty as derived from an earlier study using the same items [20]. Intervention and control items were scattered across cases, i.e. some cases did not contain any intervention/control item (these cases referred to important teaching content that was, however, not targeted in this study) while other cases contained up to five intervention/control items. All analyses (see below) were carried out on the (aggregated) item level, irrespective of the cases in which they had been embedded.

Each of the 28 intervention/control items was presented in two out of the 10 e-seminars during the learning phase. As a result, any student – regardless of group assignment – was exposed to 14 intervention (video) items in two e-seminars and to 14 control (text) items in two different e-seminars. During the final week of term, students took a formative exit exam consisting of these 28 items that had been arranged into four new cases containing 6 or 8 key features each. Again, cases were only used to provide a structure to accommodate the items, and analyses were done on the item level. A formative retention test was taken 6 months later.

Given the impact of context and format on exam performance with regard to the learning environment [21], presentation of items in the exit exam and retention test was balanced in that seven out of 14 intervention (video) items for both groups were presented as video items in these exams while the other seven were presented as text items and vice versa. This was done to address the second aim of the study outlined above. Item assignment to presentation format was balanced by item difficulty as derived from the previous study [20]. Thus, from the outset, item difficulty was expected to be similar for the 14 intervention and control items in the two study groups as well as for the 7 video and the 7 text items derived from these (respectively) in the exit exam and retention test. As a consequence, any differences in student performance were more likely to be caused by the intervention than by pre-existing differences in item difficulties.

### Data analysis

Descriptive analyses involved calculation of student mean scores achieved for intervention and control items in the three exams as well as in the first and second occurrence of an item in an e-seminar. The primary outcome for this study was the within-subject difference in percent scores in the exit exam for those 14 items that had been presented in video format during the learning phase compared to those 14 items that had been presented in text format. The difference was assessed in a paired T-test. Based on an expected sample size of 90 and the results of a previous study [20], the study was adequately powered (1 – β = 0.8) to detect a performance difference between video and text items of 8% on an α level of 5%. Performance on items in the retention test was assessed as a function of the number of correct retrievals for the same items during e-seminars and the exit exam. Performance differences as a function of presentation format during the learning phase and the formative exams was assessed using Friedman and Wilcoxon tests. According to the statistical analysis plan, an additional Bayesian approach was scheduled in case one of these analyses yielded a non-significant result. The Bayes factor was to be calculated based on the assumption that the effect of using videos instead of text would be half the size of the effect observed in the mentioned previous study comparing repeated testing to repeated study of clinical cases.

With regard to the third aim of this study, common errors were addressed by calculating the proportion of students making incorrect choices in e-seminars (learning phase) and the exit exam and retention test, respectively.

Statistical analysis was performed using SPSS 22.0 (SPSS Inc., Chicago, Illinois, USA) and the Bayes factor calculator provided by Dienes [25]. Data are presented as mean ± SD or percentages unless otherwise stated. Significance levels were set to *p* < 0.05. This study was approved by the local Ethics Committee (application number 22/4/15), and all participants provided written consent.

## Results

### Student characteristics

The flow of participants through the study is displayed in Fig. 1. Of the 125 students eligible for study participation, six did not provide written consent. Following exclusion of students due to missing data or contamination (due to exposure to the wrong presentation format at least once), complete data were available for 93 students (effective response rate 74.4%). The mean age of study participants was 25.8 ± 3.9 years, and 64.5% (*n* = 60) were female.

### Learning outcome

**Fig. 2:**
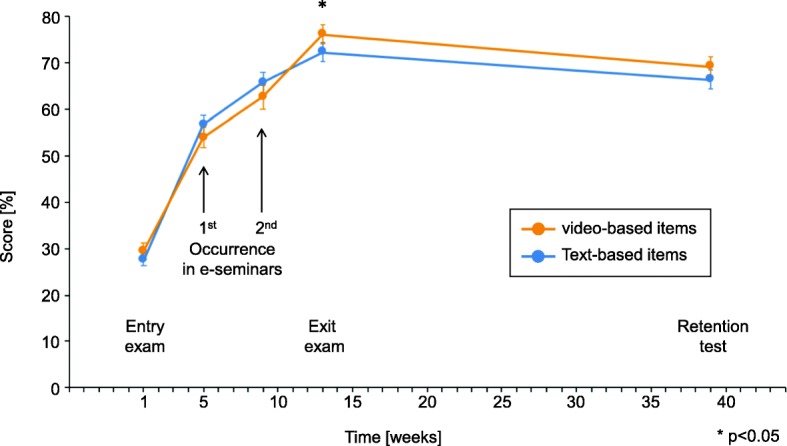
Trajectories of student performance in video-based and text-based items, respectively. The first presentation (‘1st occurrence’) of a particular item in an e-seminar occurred between week 3 and week 9; the second item presentation occurred between week 5 and week 11 (‘2nd occurrence’). In order to increase legibility, data collected during first and second occurrences, respectively, were collapsed into two data collection points although data were actually collected over a period of 6 weeks for each occurrence

Mean percent scores in the entry, exit and retention exams were 28.5 ± 13.5%, 74.3 ± 17.4% and 67.8 ± 18.5%, respectively (Fig. 2). With regard to the primary endpoint, exit exam scores in items that had been presented as videos during the learning phase were significantly higher than in text items (76.2 ± 19.4% vs. 72.4 ± 19.1%, *T* = 2.263; df = 92; *p* = 0.026). A similar but non-significant difference was found in the retention test (69.2 ± 20.2% vs. 66.4 ± 20.3%, *T* = 1.624; df = 92; *p* = 0.108). Using the mean difference of 2.8% and the corresponding standard error (1.7%) yielded a Bayes factor of 2.36, supporting the hypothesis that video items were more effective than text items. In the aggregate sample, the probability of correctly answering an item in the retention test following three failed retrievals in preceding e-seminars and the exit exam was 27.7%; the corresponding percentage for one, two and three correct retrievals were 51.3%, 70.2% and 87.5%, respectively. A descriptive analysis of all exam results on the level of individual items is presented in Additional file 1: eTable1.

### Association between presentation format during learning and in the formative exams

With regard to the different combinations between presentation format during e-seminars and in the formative exams, student performance in the four item types differed significantly for both the exit exam and the retention test (*p* < 0.001 for both). A detailed analysis revealed that, while video items were more beneficial for learning purposes than text items, the opposite was true for presentation format in the formative exams in that students achieved higher scores in text items than in video items (exit exam: 78.2 ± 18.5% vs. 70.4 ± 18.9%, *T* = 5.485; df = 92; *p* < 0.001; retention test: 72.2 ± 18.4% vs. 63.4 ± 21.2%, *T* = 5.845; df = 92; *p* < 0.001) (Table 1).

**Table 1:** Impact of alignment between presentation format during e-seminars and formative exams on student performance

Presentation format		Percent scores achieved in the formative exams	
E-seminars	Formative exam	Exit exam	Retention test
Text	Text	77.4 ± 21.0^a^	71.6 ± 20.4^d,e^
Text	Video	67.3 ± 22.2^a,b^	61.3 ± 25.4^d,f^
Video	Text	79.0 ± 20.2^b,c^	72.8 ± 23.1^f,g^
Video	Video	73.4 ± 22.3^c^	65.6 ± 22.5^e,g^

The Friedman test was significant for both the exit exam and the retention test. Different pairs of superscript letters indicate a significant difference between two groups in Wilcoxon signed-rank tests after Bonferroni correction for multiple comparisons

### Common errors observed in e-seminars and formative exams

Given the small differences between video and text items, data from the two study groups were collapsed for the analysis of common errors. Table 2 presents proportions of correct and incorrect answers averaged across the two e-seminars, the exit exam and the retention test. Frequent errors included mechanical ventilation for isolated hypoxemia (24.2%), thrombolytic treatment without confirmation of a diagnosis of pulmonary embolism (17.7%), oxygen supplementation for CO_2_ poisoning (16.9%), ECG diagnosis of a conduction block instead of atrial fibrillation (13.7%), rapid sodium supplementation for chronic hyponatremia (11.0%), and medical or surgical treatment for hyperthyroidism in a patient still receiving amiodarone (17.2%). A detailed analysis by item type (video or text) and data collection point is included in Additional file 1: eTable 2.

**Table 2:** Proportions of correct and incorrect answers for the 28 key feature items, calculated across the two e-seminars, and exit exam and the retention test

Diseases	Key features (Items)	Proportion of correct answers	Frequent or relevant incorrect answers (proportion)
Pulmonary embolism	Diagnosis of *pulmonary embolism*	58.6%	Acute coronary syndrome (8.0%); aortic dissection (3.9%)
	*Wells Score* to assess likelihood of PE	74.0%	Assessment of hemodynamic stability (5.2%)
	*Thorax CT scan* to confirm PE in a patient with high clinical probability	50.0%	Thrombolysis without confirmation (17.7%); other imaging (9.7%); D-dimer testing (9.7%)
	*Right ventricular strain* for risk stratification	51.9%	
	*Fibrinolysis* for unstable pulmonary embolism	66.6%	Other medical treatment (10.2%)
Arterial hypertension	Diagnosis of *secondary hypertension*	49.6%	Essential hypertension (11.5%); cardiac disease (10.4%)
	Diagnosis of *diastolic dysfunction*	17.5%	Other myocardial disease (60.8%)
	*Discontinuation of ACE inhibitors* due to typical cough	93.7%	
Hyponatremia	*Hospital admission* for chronic symptomatic hyponatremia	70.5%	Rapid sodium supplementation (11.0%)
	*Thiazide diuretics* as cause of hyponatremia	83.0%	Hypovolemia (3.4%)
	Diagnosis of *central pontine myelinolysis* following rapid sodium supplementation	64.9%	Cerebral oedema (13.6%)
Atrial fibrillation	*Orthostatic challenge* after syncope	72.5%	Tilt testing (22.8%)
	ECG diagnosis of *tachyarrhythmia*	61.8%	Conduction blocks (13.7%); myocardial infarction (1.9%)
	*CHA*_*2*_*DS*_*2*_*-VASc score* for anticoagulation	83.6%	
Lupus erythematosus	Diagnosis of *nephrotic syndrome*	80.3%	Nephritic syndrome (8.0%)
	Diagnosis of *systemic lupus erythematosus*	83.8%	Non-specific glomerulonephritis (4.3%)
	Renal *biopsy* to confirm lupus nephritis	79.8%	Imaging (4.6%)
COPD	Diagnosis of *COPD*	91.1%	
	Confirmation of COPD by *FEV*_*1*_*/VC < 70%*	64.7%	FEV_1_ (13.6%); FEV_1_/VC % predicted (15.8%)
	*ABG analysis* for suspected CO_2_ intoxication	67.2%	Imaging (7.2%)
	Treatment of CO_2_ intoxication by *non-invasive ventilation*	53.3%	Oxygen (16.9%); CPAP (11.4%); buffering (6.7%)
Pneumonia	Diagnosis of *left apical pneumonia* in a chest X-ray	72.8%	Incorrect or no localisation (23.3%)
	Decision on hospitalisation based on the *CRB-65 score*	75.9%	Decision based on additional laboratory tests (5.6%)
Hyperthyroidism	Diagnosis of *hyperthyroidism* from lab results	88.8%	Hypothyroidism (4.1%)
	*Stopping amiodarone* in a pt. with hyperthyroidism	66.2%	other medical (11.4%) or surgical (5.7%) treatment
Pulmonary fibrosis	Diagnosis of *pulmonary fibrosis*	53.8%	Pulmonary oedema (12.5%); pneumonia (9.4%)
	*Amiodarone* as cause of pulmonary fibrosis	78.9%	Atrial fibrillation (4.8%)
	Indication for *long-term oxygen therapy*	47.6%	Mechanical ventilation (24.2%)

*ABG* arterial blood gases, *ACE* angiotensin converting enzyme, *CAD* coronary artery disease, *CHA*_*2*_*DS*_*2*_*-VASc* congestive heart failure, hypertension, age ≥75 (doubled), diabetes, stroke (doubled), vascular disease, age 65–74, and sex category (female), CO_*2*_ carbon dioxide, *COPD* chronic obstructive pulmonary disease, *CPAP* continuous positive airway pressure, *CRB* confusion/respiratory rate/blood pressure, *CT* computed tomography, *ECG* electrocardiogram, *FEV*_*1*_ forced expiratory volume in 1 s, *NIV* non-invasive ventilation, *PAD* peripheral artery disease, *PE* pulmonary embolism, *VC* vital capacity

Italics indicate the key feature assessed in each item; answers that were frequently entered instead of these are shown in the last column of the table

## Discussion

This randomised cross-over study yielded two main findings. First, repeated testing with video-based key feature questions elicited higher short-term learning outcomes than testing with text-based questions regarding diagnostic accuracy and initial management issues with only weak evidence in the long-term. Second, we found a high prevalence of erroneous beliefs in medical students irrespective of item format (video or text). These misconceptions have the potential to negatively impact the quality of patient care unless corrected before graduation.

### *Choosing Wisely* – a perspective on medical education

Clinical reasoning abilities are essential for competent and reflective physicians. Undergraduate medical education needs to equip students with a solid knowledge base and train them to apply that knowledge in a given clinical context. The *Choosing Wisely* recommendations emerged from clinical reasoning processes. Thus, the initiative could serve as a paradigm of quality assurance in clinical care that should be addressed in medical education. Albeit not having been specifically designed to match any of the top five lists, some of the key feature questions embedded in the patient cases presented to our students alluded to current *Choosing Wisely* recommendations. For example, the lists of both the American College of Chest Physicians and the American College of Emergency Physicians cover the treatment of pulmonary embolism, and our results (Table 2) show that some fourth-year medical students struggle with basic concepts such as confirming the diagnosis before initiating treatment or skipping D-dimer testing in patients with a high probability of pulmonary embolism. Our data also shed light on other misconceptions that may have fatal consequences (e.g. sole oxygen supplementation without ventilator support in CO_2_ poisoning). Medical education research preferentially focuses on correct responses and aggregate scores. In the light of the *Choosing Wisely* campaign, a more thorough assessment of misconceptions and frequent errors appears promising in that it could guide improvements in the way clinical reasoning is being taught. This process should be informed by educational research. The present study suggests that repeated testing with key feature questions is effective in enhancing clinical reasoning performance.

### Test-enhanced learning for clinical reasoning: a proof of concept

Since the introduction of test-enhanced learning to the field of medical education research, a number of studies on undergraduate medical [17], dental [26] and nursing [27] education have confirmed that repeated testing can be used to enhance retention of knowledge and skills relevant to preclinical [28] and clinical [29] medicine. The performance retention rates observed in our study are close to or exceed the upper bound of results reported in previous studies. In two prospective randomised trials involving medical students and residents, respectively, percent scores in retention tests 6 months after a test-based intervention approached 40% [16, 29]. Our study adds to the growing body of literature supporting the principle of test-enhanced learning in medical education by demonstrating that a resource-saving, computer-based intervention can have a beneficial impact on outcomes related to clinical reasoning.

### Use of videos in medical education

There is a strong rationale for using audio-visual material in higher education as learning resources that are more closely aligned to the context in which knowledge will be applied is likely to produce more favourable learning outcomes [21]. However, high-quality studies on the effective use of videos in medical education are scarce, and only very few have assessed their potential to enhance clinical reasoning. If anything, the available evidence is apt to raise concerns regarding the validity of effectiveness claims. While video triggers are preferred by students in the context of problem-based learning [30, 31], recent research suggests that video-based problem-based learning cases may disrupt deep critical thinking [32], and that increased authenticity of instructional formats does not necessarily improve clinical reasoning performance in learners [33]. Our current study lends support to the notion that, when used for key feature testing, videos can enhance short-term retention of knowledge relevant to clinical reasoning. Our finding of a greater performance increase between the first e-seminar and the exit exam for video than for text items may be explained by the fact that videos are most effective in situations where sensory information (that cannot be provided in a text) helps to solve a problem [34]. For example, in a patient with suspected pulmonary fibrosis, the video contained an audio recording of the auscultation. Students watching the video were less likely to incorrectly diagnose this patient as having pulmonary oedema and more likely to spot the right diagnosis (Additional file 1: eTable 2). On the other hand, the wealth of information contained in videos increases cognitive load for learners [35] and may also increase the effort associated with retrieval in the context of test-enhanced learning. Our data support the theory that effortful retrieval produces superior retention [14]. The results of the Bayesian analysis with regard to long-term retention can be used to inform future studies in terms of priors for future analyses. This is particularly helpful when evaluating costly interventions.

### Strengths and limitations

Test-enhanced learning is an emerging concept in medical education, and this is one of the first studies using video-based key feature questions to enhance clinical reasoning performance. The questions used during the learning phase and in formative exams referred to highly prevalent or important clinical problems, and all questions were piloted extensively [20]. The response rate was favourable, and randomisation minimised any effects potentially related to self-selection of students.

Despite these strengths, the generalisability of our findings is limited by the monocentric nature of the study. Given that the cases used focussed on internal medicine, we cannot draw conclusions on the potential of test-enhanced learning with key feature questions for other medical specialties. Finally, this study did not assess whether repeated testing with key feature questions impacts on actual student performance in the clinical setting. Although one study suggests such a link [36], more research is needed to establish a causal relation between repeated testing and more favourable patient outcomes.

## Conclusions

Repeated video-based key feature testing was associated with significantly better clinical reasoning performance compared to text-based testing in the short term. However, student responses revealed a high prevalence of erroneous beliefs that – if applied to the clinical context – are potentially harmful to patients. Efforts to improve clinical reasoning training in medical education are warranted, and the *Choosing Wisely* campaign lends itself to being part of this process aimed at supporting students to become reflective and competent physicians.

## Additional files

Additional file 1: **Methods.** Description and example of a key feature question. **eTable 1.** Proportions of correct answers for the 28 key feature items in the exit exam and the retention test. **eTable 2.** Frequent or relevant incorrect answers (proportion). (DOCX 59 kb)
