# Supplementary material for: How can we teach medical students to choose wisely? A randomised controlled cross-over study of video- versus text-based case scenarios
Source: BMC Med. 2018 Jul 6;16:107. doi: 10.1186/s12916-018-1090-y (PMC6034339; doi:10.1186/s12916-018-1090-y)
Supplement: Supplementary file 2 — Methods. Description and example of a key feature question. eTable 1. Proportions of correct answers for the 28 key feature items in the exit exam and the retention test. eTable 2. Frequent or relevant incorrect answers (proportion). (DOCX 59 kb) [file 12916_2018_1090_MOESM1_ESM.docx]

**How can we teach medical students to choose wisely?**

**A randomized controlled cross-over study**

**of video- versus text-based case scenarios**

Online supplement

**Methods:** Description and example of a key feature question 2

**Results – eTable 1**: Proportions of correct answers for the 28 key feature items in the exit exam and the retention test. 4

**Results – eTable 2:** Frequent or relevant incorrect answers (proportion) 5

**Methods**

Example of a key feature question

All patient cases used in our study were made up of five sections containing clinical information. At the end of each section, students were asked to answer a key feature question (i.e., item). Unlike multiple or single choice questions, key feature questions do not require students to *recognize* the correct answer from a short list of items. Instead, students need to *produce* an answer and enter it into a free-text box. The software used to facilitate key feature questions is fitted with a list of diagnoses, diagnostic tests and therapeutic interventions. This list is called a ‘long menu’. Once a student has entered three or more letters of the answer he/she had in mind, all items from the long menu containing the same letters (in the same order) are being displayed, and the student can choose the one he/she was looking for. Accordingly, teachers need to take great care to add any items to the long menu that might be considered correct. In order to be able to detect misconceptions, incorrect answers (‘distractors’) need to be included as well.

Since the correct answer to a key feature question cannot be picked from a pre-defined selection of options but students have to actively look for the desired answer, key feature questions and other open-ended formats are considered to be ‘production tests’ as opposed to ‘recognition tests’ such as multiple choice questions. In the context of test-enhanced learning, production tests should be preferred as they increase cognitive load, thereby enhancing the beneficial effect of knowledge retrieval on long-term retention.

An example of a key feature question used in our study is given on the next page.

Key feature (‘item’): Suspected pulmonary fibrosis in a patient called “Mr. Teschke” (fictional name) with shortness of breath, inspiratory crackles and a history of amiodarone medication

Vignette (presented on the computer screen):

“Mr. Teschke is a 74 year-old patient who suffered from a stroke as a result of atrial fibrillation two years ago. His current medication includes metoprolol, ramipril, simvastatin, hydrochlorothiazide, allopurinol, amiodarone, and warfarin. Today, he reports to your surgery due to increasing shortness of breath that he has been feeling for ‘a while now’. He tells you that he had hoped the symptoms would abate. Unfortunately, he now feels out of breath almost all of the time. On the physical examination of the thorax, you note ubiquitous inspiratory crackles.”

Question (presented on the computer screen, followed by a blank write-in field):

“What is the most likely diagnosis?”

Once a student has entered the first letters of the desired response, all available options from the long menu containing these letters are displayed. Upon making a choice, the student is directed to the next screen where he/she is provided with detailed feedback to the preceding question. In addition, all long menu options that were pre-defined as correct answers are displayed so that the student can check the accuracy of his/her answer. Following this, the case continues to the next key feature question (i.e., item). Students can navigate back within cases, but they cannot change answers they have entered.

**eTable 1:** **Proportions of correct answers for the 28 key feature items in the exit exam and the retention test.**

* p<0.05 in a χ^2^ test comparing video and text items. AGB, arterial blood gases; ACE, angiotensin converting enzyme; CAD, coronary artery disease; CO_2_, carbon dioxide; COPD, chronic obstructive pulmonary disease; CRB, confusion/respiratory rate/blood pressure; CT, computed tomography; ECG, electrocardiogram; FEV_1_, forced expiratory volume in 1 second; NIV, non-invasive ventilation; PAD, peripheral artery disease; PE, pulmonary embolism; VC, vital capacity.

| **Diseases** | **Key features (Items)** | **Exit exam** | | **Retention test** | |
| --- | --- | --- | --- | --- | --- |
|  |  | **Video items** | **Text items** | **Video items** | **Text items** |
| Pulmonary embolism | Diagnosis of pulmonary embolism | 77.8 | 81.3 | 48.9 | 68.8 |
|  | Wells Score before D-dimer testing | 86.7 | 81.3 | 77.8 | 70.8 |
|  | Thorax CT scan to confirm PE | 48.9 | 56.3 | 42.2 | 47.9 |
|  | Right ventricular strain for risk stratification | 62.2 | 64.6 | 57.8 | 54.2 |
|  | Fibrinolysis for unstable pulmonary embolism | 73.3 | 68.8 | 55.6 | 62.5 |
| Arterial hypertension | Diagnosis of secondary hypertension | 66.7 | 60.4 | 66.7 | 64.6 |
|  | Diagnosis of diastolic dysfunction | 20.0 | 27.1 | 22.2 | 25.0 |
|  | Diagnosis of ACE inhibitor cough | 100.0 | 91.7* | 95.6 | 95.8 |
| Hyponatremia | Hospital admission for hyponatremia | 82.2 | 64.6 | 71.1 | 60.4 |
|  | Thiazide diuretics as cause of hyponatremia | 93.3 | 85.4 | 77.8 | 85.4 |
|  | Diagnosis of central pontine myelinolysis | 91.1 | 79.2 | 64.4 | 54.2 |
| Atrial fibrillation | Orthostatic challenge after syncope | 80.0 | 72.9 | 64.4 | 64.6 |
|  | ECG diagnosis of tachyarrhythmia | 75.6 | 75.0 | 75.6 | 64.6 |
|  | CHA_2_DS_2_-VASc score for anticoagulation | 91.1 | 89.6 | 91.1 | 83.3 |
| Lupus erythematosus | Diagnosis of Nephrotic Syndrome | 81.3 | 91.1 | 68.8 | 64.4 |
|  | Diagnosis of systemic lupus erythematosus | 89.6 | 93.3 | 93.8 | 97.8 |
|  | Renal biopsy to confirm lupus nephritis | 83.3 | 77.8 | 83.3 | 91.1 |
| COPD | Diagnosis of COPD | 91.7 | 91.1 | 87.5 | 88.9 |
|  | Confirmation of COPD by FEV_1_/VC<70% | 72.9 | 57.8 | 62.5 | 46.7 |
|  | ABG analysis for suspected CO_2_ intoxication | 66.7 | 71.1 | 70.8 | 68.9 |
|  | Treatment of CO_2_ intoxication by NIV | 87.5 | 88.9 | 72.9 | 73.3 |
| Pneumonia | Diagnosis of pneumonia in a chest X-ray | 62.5 | 46.7 | 54.2 | 53.3 |
|  | CRB-65 score for hospital admission | 58.3 | 62.2 | 50.0 | 33.3 |
| Hyperthyroidism | Diagnosis of hyperthyroidism from lab results | 85.4 | 88.9 | 81.3 | 91.1 |
|  | Stopping amiodarone in a pt. with hyperthyroidism | 79.2 | 75.6 | 83.3 | 71.1 |
| Pulmonary fibrosis | Diagnosis of pulmonary fibrosis | 72.9 | 62.2 | 77.1 | 62.2 |
|  | Amiodarone as cause of pulmonary fibrosis | 87.5 | 88.9 | 91.7 | 84.4 |
|  | Indication for long-term oxygen therapy | 64.6 | 33.3* | 45.8 | 33.3 |

**eTable 2:** **Frequent or relevant incorrect answers (proportion)**

Categorized frequent or relevant incorrect answers given by the students for the key feature items, split up by presentation format in learning phase (1^st^ and 2^nd^ occurrence in the e-seminars) as well as in the formative exams (exit exam and retention test). Example: Students working on video items in the learning phase were more likely to correctly diagnose a pulmonary fibrosis (77.1%) instead of choosing the incorrect answer ‘pulmonary edema’ (2.1%) in the retention test compared to students working on text items in the learning phase (62.2% and 20%, respectively).

|  | | **Presentation format video in learning phase** | | | | **Presentation format text in learning phase** | | | |
| --- | --- | --- | --- | --- | --- | --- | --- | --- | --- |
|  |  | **learning phase** | | **formative exams** | | **learning phase** | | **formative exams** | |
|  |  | **1^st^** | **2^nd^** | **Exit exam** | **Retention test** | **1^st^** | **2^nd^** | **Exit exam** | **Retention test** |
| **Pulmonary embolism** | **Diagnosis of pulmonary embolism** | | | | | | | | |
|  | wrong answer (other) | 18.2 | 29.3 | 22.2 | 48.9 | 9.1 | 14.9 | 18.8 | 29.2 |
|  | correct answer | 27.3 | 39 | 77.8 | 48.9 | 50 | 70.2 | 81.3 | 68.8 |
|  | acute coronary syndrome | 27.3 | 0 | 0 | 0 | 36.4 | 0 | 0 | 2.1 |
|  | aortic dissection | 27.3 | 0 | 0 | 0 | 4.5 | 0 | 0 | 0 |
|  | heart failure | 0 | 7.3 | 0 | 2.2 | 0 | 6.4 | 0 | 0 |
|  | cyanosis | 0 | 14.6 | 0 | 0 | 0 | 2.1 | 0 | 0 |
|  | anemia | 0 | 9.8 | 0 | 0 | 0 | 6.4 | 0 | 0 |
|  | **Wells Score to assess likelihood of PE** | | | | | | | | |
|  | wrong answer (other) | 29.5 | 14.6 | 8.9 | 20 | 25 | 10.6 | 12.5 | 27.1 |
|  | correct answer | 54.5 | 70.7 | 86.7 | 77.8 | 70.5 | 78.7 | 81.3 | 70.8 |
|  | haemodynamic stability | 11.4 | 12.2 | 2.2 | 0 | 4.5 | 6.4 | 6.3 | 0 |
|  | imaging | 4.5 | 2.4 | 2.2 | 2.2 | 0 | 4.3 | 0 | 2.1 |
|  | **Thorax CT scan to confirm PE** | | | | | | | | |
|  | wrong answer (other) | 11.4 | 9.8 | 4.4 | 6.7 | 9.1 | 4.3 | 8.3 | 12.5 |
|  | correct answer | 47.7 | 48.8 | 48.9 | 42.2 | 56.8 | 51.1 | 56.3 | 47.9 |
|  | other imaging | 2.3 | 12.2 | 11.1 | 15.6 | 9.1 | 8.5 | 8.3 | 8.3 |
|  | therapeutic anticoagulation | 15.9 | 2.4 | 8.9 | 4.4 | 2.3 | 27.7 | 4.2 | 2.1 |
|  | systemic thrombolysis | 20.5 | 26.8 | 20 | 13.3 | 11.4 | 0 | 4.2 | 18.8 |
|  | D-dimer testing | 2.3 | 0 | 6.7 | 17.8 | 11.4 | 8.5 | 18.8 | 10.4 |
|  | **Right ventricular strain for risk stratification** | | | | | | | | |
|  | wrong answer (other) | 56.8 | 46.3 | 24.4 | 31.1 | 43.2 | 27.7 | 14.6 | 31.3 |
|  | correct answer | 38.6 | 39 | 62.2 | 57.8 | 43.2 | 53.2 | 64.6 | 54.2 |
|  | other diagnostic tests (non-laboratory) | 2.3 | 12.2 | 11.1 | 8.9 | 9.1 | 6.4 | 12.5 | 10.4 |
|  | laboratory testing | 2.3 | 2.4 | 2.2 | 2.2 | 4.5 | 12.8 | 8.3 | 4.2 |
|  | **Fibrinolysis for unstable pulmonary embolism** | | | | | | | | |
|  | wrong answer (other) | 20.5 | 34.1 | 11.1 | 28.9 | 11.4 | 17 | 16.7 | 25 |
|  | correct answer | 68.2 | 56.1 | 73.3 | 55.6 | 75 | 72.3 | 68.8 | 62.5 |
|  | other medical treatment | 6.8 | 4.9 | 11.1 | 13.3 | 9.1 | 8.5 | 14.6 | 12.5 |
|  | thrombectomy | 4.5 | 4.9 | 4.4 | 2.2 | 4.5 | 2.1 | 0 | 0 |
| **Arterial hypertension** | **Diagnosis of secondary hypertension** | | | | | | | | |
|  | wrong answer (other) | 27.9 | 25 | 28.9 | 17.8 | 4.3 | 17.4 | 22.9 | 16.7 |
|  | correct answer | 20.9 | 34.1 | 66.7 | 66.7 | 52.2 | 28.3 | 60.4 | 64.6 |
|  | myocardial disease | 18.6 | 2.3 | 0 | 2.2 | 32.6 | 2.2 | 0 | 0 |
|  | valve disease | 25.6 | 0 | 0 | 0 | 2.2 | 0 | 0 | 0 |
|  | hypertension other than secondary | 7 | 13.6 | 4.4 | 13.3 | 8.7 | 8.7 | 16.7 | 18.8 |
|  | kidney disease | 0 | 25 | 0 | 0 | 0 | 43.5 | 0 | 0 |
|  | **Diagnosis of diastolic dysfunction** | | | | | | | | |
|  | wrong answer (other) | 16.3 | 36.4 | 13.3 | 24.4 | 23.9 | 26.1 | 12.5 | 20.8 |
|  | correct answer | 0 | 9.1 | 20 | 22.2 | 10.9 | 23.9 | 27.1 | 25 |
|  | myocardial disease | 83.7 | 54.5 | 66.7 | 53.3 | 65.2 | 50 | 60.4 | 54.2 |
|  | **Discontinuation of ACE inhibitors due to typical cough** | | | | | | | | |
|  | wrong answer (other) | 0 | 0 | 0 | 2.2 | 6.5 | 8.5 | 8.3 | 4.2 |
|  | correct answer | 93 | 97.6 | 100 | 95.6 | 87 | 89.4 | 91.7 | 95.8 |
|  | AT1 inhibitor | 7 | 2.4 | 0 | 2.2 | 6.5 | 2.1 | 0 | 0 |
| **Hyponatremia** | **Hospital admission for hyponatremia** | | | | | | | | |
|  | wrong answer (other) | 19.5 | 5.1 | 8.9 | 6.7 | 19.1 | 5 | 18.8 | 8.3 |
|  | correct answer | 65.9 | 92.3 | 82.2 | 71.1 | 57.4 | 75 | 64.6 | 60.4 |
|  | sodium supplementation | 12.2 | 0 | 8.9 | 11.1 | 14.9 | 12.5 | 16.7 | 10.4 |
|  | discontinuation of diuretics | 2.4 | 2.6 | 0 | 11.1 | 8.5 | 7.5 | 0 | 20.8 |
|  | **Thiazide diuretics as cause of hyponatraemia** | | | | | | | | |
|  | wrong answer (other) | 7.3 | 5.1 | 2.2 | 15.6 | 8.5 | 7.5 | 10.4 | 4.2 |
|  | correct answer | 75.6 | 92.3 | 93.3 | 77.8 | 68.1 | 87.5 | 85.4 | 85.4 |
|  | hypovolaemia | 14.6 | 0 | 0 | 0 | 10.6 | 2.5 | 0 | 0 |
|  | other medication | 2.4 | 2.6 | 4.4 | 6.7 | 12.8 | 2.5 | 4.2 | 10.4 |
|  | **Diagnosis of central pontine myelinolysis** | | | | | | | | |
|  | wrong answer (other) | 24.4 | 7.7 | 6.7 | 24.4 | 23.4 | 10 | 12.5 | 29.2 |
|  | correct answer | 36.6 | 74.4 | 91.1 | 64.4 | 42.6 | 77.5 | 79.2 | 54.2 |
|  | cerebral oedema | 34.1 | 15.4 | 2.2 | 8.9 | 21.3 | 12.5 | 4.2 | 12.5 |
|  | hypernatraemia | 4.9 | 2.6 | 0 | 2.2 | 12.8 | 0 | 4.2 | 4.2 |
| **Atrial fibrillation** | **Orthostatic challenge after syncope** | | | | | | | | |
|  | wrong answer (other) | 0 | 0 | 2.2 | 6.7 | 4.3 | 4.5 | 4.2 | 6.3 |
|  | correct answer | 77.3 | 79.5 | 80 | 64.4 | 65.2 | 77.3 | 72.9 | 64.6 |
|  | tilt testing | 20.5 | 18.2 | 17.8 | 28.9 | 28.3 | 18.2 | 20.8 | 29.2 |
|  | blood pressure measurements | 2.3 | 2.3 | 0 | 0 | 2.2 | 0 | 2.1 | 0 |
|  | **ECG diagnosis of tachyarrhythmia** | | | | | | | | |
|  | wrong answer (other) | 25 | 41.5 | 13.3 | 13.3 | 30.4 | 29.8 | 14.6 | 14.6 |
|  | correct answer | 47.7 | 39 | 75.6 | 75.6 | 47.8 | 66 | 75 | 64.6 |
|  | conduction abnormalities | 25 | 19.5 | 8.9 | 11.1 | 19.6 | 4.3 | 6.3 | 16.7 |
|  | myocardial infarction | 2.3 | 0 | 2.2 | 0 | 2.2 | 0 | 4.2 | 4.2 |
|  | **CHA_2_DS_2_-VASc score for anticoagulation** | | | | | | | | |
|  | wrong answer (other) | 34.1 | 17.9 | 8.9 | 8.9 | 21.3 | 15 | 10.4 | 16.7 |
|  | correct answer | 65.9 | 82.1 | 91.1 | 91.1 | 78.7 | 85 | 89.6 | 83.3 |
| **Lupus erythematosus** | **Diagnosis of Nephrotic Syndrome** | | | | | | | | |
|  | wrong answer (other) | 9.5 | 9.3 | 10.4 | 14.6 | 9.5 | 0 | 2.2 | 20 |
|  | correct answer | 81 | 88.4 | 81.3 | 68.8 | 73.8 | 97.4 | 91.1 | 64.4 |
|  | Nephritic syndrome | 7.1 | 2.3 | 8.3 | 16.7 | 4.8 | 2.6 | 4.4 | 15.6 |
|  | Lupus erythematosus | 2.4 | 0 | 0 | 0 | 0 | 0 | 0 | 0 |
|  | Glomerulonephritis | 0 | 0 | 0 | 0 | 11.9 | 0 | 2.2 | 0 |
|  | **Diagnosis of systemic lupus erythematosus** | | | | | | | | |
|  | wrong answer (other) | 35.7 | 9.3 | 8.3 | 6.3 | 7.1 | 0 | 2.2 | 2.2 |
|  | correct answer | 40.5 | 90.7 | 89.6 | 93.8 | 64.3 | 97.4 | 93.3 | 97.8 |
|  | Glomerulonephritis | 14.3 | 0 | 0 | 0 | 14.3 | 2.6 | 4.4 | 0 |
|  | other immunologic disease | 9.5 | 0 | 2.1 | 0 | 14.3 | 0 | 0 | 0 |
|  | **Renal biopsy to confirm lupus nephritis** | | | | | | | | |
|  | wrong answer (other) | 14.3 | 9.3 | 4.2 | 10.4 | 26.2 | 7.9 | 15.6 | 4.4 |
|  | correct answer | 64.3 | 88.4 | 83.3 | 83.3 | 61.9 | 86.8 | 77.8 | 91.1 |
|  | imaging | 9.5 | 2.3 | 4.2 | 2.1 | 9.5 | 5.3 | 2.2 | 2.2 |
|  | laboratory testing | 11.9 | 0 | 8.3 | 4.2 | 2.4 | 0 | 4.4 | 2.2 |
| **COPD** | **Diagnosis of COPD** | | | | | | | | |
|  | wrong answer (other) | 6.5 | 9.5 | 8.3 | 12.5 | 2.3 | 4.8 | 8.9 | 11.1 |
|  | correct answer | 93.5 | 90.5 | 91.7 | 87.5 | 93.2 | 92.9 | 91.1 | 88.9 |
|  | asthma | 0 | 0 | 0 | 0 | 4.5 | 2.4 | 0 | 0 |
|  | **Confirmation of COPD by FEV_1_/VC<70%** | | | | | | | | |
|  | wrong answer (other) | 4.3 | 2.4 | 10.4 | 10.4 | 2.3 | 0 | 6.7 | 8.9 |
|  | correct answer | 60.9 | 85.7 | 72.9 | 62.5 | 56.8 | 76.2 | 57.8 | 46.7 |
|  | FEV1 | 28.3 | 7.1 | 8.3 | 6.3 | 20.5 | 9.5 | 15.6 | 13.3 |
|  | FEV1/VC (% predicted) | 6.5 | 4.8 | 8.3 | 20.8 | 20.5 | 14.3 | 20 | 31.1 |
|  | **ABG analysis for suspected CO_2_ intoxication** | | | | | | | | |
|  | wrong answer (other) | 13 | 9.5 | 27.1 | 35.4 | 22.7 | 2.4 | 28.9 | 35.6 |
|  | correct answer | 71.7 | 83.3 | 62.5 | 54.2 | 72.7 | 97.6 | 46.7 | 53.3 |
|  | chest X-ray | 13 | 4.8 | 4.2 | 0 | 2.3 | 0 | 2.2 | 0 |
|  | lung function testing | 2.2 | 2.4 | 0 | 0 | 2.3 | 0 | 0 | 0 |
|  | blood cultures | 0 | 0 | 4.2 | 2.1 | 0 | 0 | 11.1 | 2.2 |
|  | other imaging | 0 | 0 | 2.1 | 8.3 | 0 | 0 | 11.1 | 8.9 |
|  | **Treatment of CO_2_ intoxication by NIV** | | | | | | | | |
|  | wrong answer (other) | 10.9 | 9.5 | 4.2 | 10.4 | 13.6 | 7.1 | 6.7 | 13.3 |
|  | correct answer | 58.7 | 64.3 | 58.3 | 50 | 43.2 | 57.1 | 62.2 | 33.3 |
|  | CPAP | 10.9 | 4.8 | 8.3 | 10.4 | 13.6 | 11.9 | 11.1 | 20 |
|  | oxygen | 10.9 | 7.1 | 22.9 | 27.1 | 15.9 | 7.1 | 17.8 | 24.4 |
|  | buffering | 8.7 | 14.3 | 6.3 | 2.1 | 9.1 | 4.8 | 2.2 | 6.7 |
|  | intubation | 0 | 0 | 0 | 0 | 4.5 | 11.9 | 0 | 2.2 |
| **Pneumonia** | **Diagnosis of pneumonia in a chest X-ray** | | | | | | | | |
|  | wrong answer (other) | 0 | 7 | 10.4 | 4.2 | 2.3 | 0 | 2.2 | 4.4 |
|  | correct answer | 78.3 | 65.1 | 66.7 | 70.8 | 88.6 | 73.7 | 71.1 | 68.9 |
|  | pneumonia, incorrect localisation | 2.2 | 25.6 | 2.1 | 0 | 2.3 | 26.3 | 0 | 0 |
|  | pneumonia, specific but incorrect | 15.2 | 2.3 | 4.2 | 4.2 | 6.8 | 0 | 8.9 | 2.2 |
|  | pneumonia, non-specific | 4.3 | 0 | 16.7 | 20.8 | 0 | 0 | 17.8 | 24.4 |
|  | **CRB-65 score for hospital admission** | | | | | | | | |
|  | wrong answer (other) | 17.4 | 14 | 8.3 | 20.8 | 27.3 | 23.7 | 11.1 | 26.7 |
|  | correct answer | 69.6 | 83.7 | 87.5 | 72.9 | 59.1 | 71.1 | 88.9 | 73.3 |
|  | laboratory testing | 13 | 2.3 | 4.2 | 6.3 | 13.6 | 5.3 | 0 | 0 |
| **Hyper-thyroidism** | **Diagnosis of hyperthyroidism from lab results** | | | | | | | | |
|  | wrong answer (other) | 6.4 | 4.3 | 8.3 | 8.3 | 4.5 | 2.3 | 2.2 | 2.2 |
|  | correct answer | 89.4 | 87 | 85.4 | 81.3 | 93.2 | 95.5 | 88.9 | 91.1 |
|  | hypothyroidism | 4.3 | 4.3 | 6.3 | 4.2 | 2.3 | 2.3 | 4.4 | 4.4 |
|  | latent hyperthyroidism | 0 | 4.3 | 0 | 6.3 | 0 | 0 | 4.4 | 2.2 |
|  | **Stopping amiodarone in a pt. with hyperthyroidism** | | | | | | | | |
|  | wrong answer (other) | 17 | 15.2 | 10.4 | 10.4 | 25 | 13.6 | 15.6 | 13.3 |
|  | correct answer | 42.6 | 84.8 | 79.2 | 83.3 | 20.5 | 70.5 | 75.6 | 71.1 |
|  | surgical treatment | 17 | 0 | 6.3 | 0 | 13.6 | 4.5 | 0 | 4.4 |
|  | medical treatment | 21.3 | 0 | 4.2 | 6.3 | 36.4 | 6.8 | 8.9 | 8.9 |
|  | RJT | 2.1 | 0 | 0 | 0 | 4.5 | 4.5 | 0 | 2.2 |
| **Pulmonary fibrosis** | **Diagnosis of pulmonary fibrosis** | | | | | | | | |
|  | wrong answer (other) | 19 | 2.3 | 8.3 | 4.2 | 4.8 | 5.3 | 4.4 | 4.4 |
|  | correct answer | 26.2 | 48.8 | 72.9 | 77.1 | 16.7 | 57.9 | 62.2 | 62.2 |
|  | cardiac disease | 9.5 | 9.3 | 0 | 2.1 | 16.7 | 2.6 | 0 | 0 |
|  | pneumonia | 9.5 | 4.7 | 4.2 | 4.2 | 31 | 5.3 | 11.1 | 6.7 |
|  | pulmonary edema | 16.7 | 2.3 | 6.3 | 2.1 | 21.4 | 15.8 | 17.8 | 20 |
|  | other pulmonary disease | 19 | 32.6 | 8.3 | 10.4 | 9.5 | 13.2 | 4.4 | 6.7 |
|  | **Amiodarone as cause of pulmonary fibrosis** | | | | | | | | |
|  | wrong answer (other) | 19 | 14 | 10.4 | 8.3 | 23.8 | 13.2 | 8.9 | 15.6 |
|  | correct answer | 57.1 | 72.1 | 87.5 | 91.7 | 59.5 | 86.8 | 88.9 | 84.4 |
|  | atrial fibrillation | 14.3 | 11.6 | 2.1 | 0 | 9.5 | 0 | 2.2 | 0 |
|  | (occupational) exposition towards cigarette smoke and fumes | 9.5 | 2.3 | 0 | 0 | 7.1 | 0 | 0 | 0 |
|  | **Indication for long-term oxygen therapy** | | | | | | | | |
|  | wrong answer (other) | 31 | 16.3 | 22.9 | 27.1 | 16.7 | 23.7 | 35.6 | 51.1 |
|  | correct answer | 47.6 | 53.5 | 64.6 | 47.9 | 54.8 | 42.1 | 33.3 | 35.6 |
|  | mechanical ventilation | 21.4 | 30.2 | 12.5 | 25 | 28.6 | 34.2 | 31.1 | 13.3 |
